# An inverse association between the Mediterranean diet and bladder cancer risk: a pooled analysis of 13 cohort studies

**DOI:** 10.1007/s00394-019-01907-8

**Published:** 2019-02-08

**Authors:** Willem J. A. Witlox, Frits H. M. van Osch, Maree Brinkman, Sylvia Jochems, Maria E. Goossens, Elisabete Weiderpass, Emily White, Piet A. van den Brandt, Graham G. Giles, Roger L. Milne, Inge Huybrechts, Hans-Olov Adami, Bas Bueno-de-Mesquita, Anke Wesselius, Maurice P. Zeegers

**Affiliations:** 1 Department of Complex Genetics and Epidemiology, NUTRIM School for Nutrition and Translational Research in Metabolism, Maastricht University, Universiteitssingel 40, 6200 MD Maastricht, The Netherlands; 2 Institute of Cancer and Genomic Sciences, University of Birmingham, Birmingham, UK; 3 Department of Clinical Studies and Nutritional Epidemiology, Nutrition Biomed Research Institute, Melbourne, Australia; 4 Department of General Practice, Katholieke Universiteit Leuven, ACHG-KU Leuven, Kapucijnenvoer 33, Blok J, bus 7001, 3000 Leuven, Belgium; 5 Department of Medical Epidemiology and Biostatistics, Karolinska Institutet, Stockholm, Sweden; 6 Department of Research, Cancer Registry of Norway, Institute of Population-Based Cancer Research, Oslo, Norway; 7 Genetic Epidemiology Group, Folkhälsan Research Center and Faculty of Medicine, University of Helsinki, Helsinki, Finland; 8 Department of Community Medicine, Faculty of Health Sciences, University of Tromsø, The Arctic University of Norway, Tromsø, Norway; 9 Fred Hutchinson Cancer Research Center, Seattle, WA USA; 10 Department of Epidemiology, Schools for Oncology and Developmental Biology and Public Health and Primary Care, Maastricht University Medical Centre, Maastricht, The Netherlands; 11 Cancer Epidemiology and Intelligence Division, Cancer Council Victoria, Melbourne, VIC Australia; 12 Centre for Epidemiology and Biostatistics, School of Population and Global Health, The University of Melbourne, Melbourne, VIC Australia; 13 International Agency for Research on Cancer (IARC), World Health Organization, Lyon, France; 14 Department of Medical Epidemiology and Biostatistics, Karolinska Institutet, Stockholm, Sweden; 15 Clinical Effectiveness Research Group, Institute of Health and Society, University of Oslo, Oslo, Norway; 16 Department for Determinants of Chronic Diseases (DCD), National Institute for Public Health and the Environment (RIVM), BA Bilthoven, The Netherlands; 17 Department of Gastroenterology and Hepatology, University Medical Centre, Utrecht, The Netherlands; 18 Department of Epidemiology and Biostatistics, The School of Public Health, Imperial College London, St Mary’s Campus, London, UK; 19 Department of Social & Preventive Medicine, Faculty of Medicine, University of Malaya, Kuala Lumpur, Malaysia; 20 CAPHRI School for Public Health and Primary Care, University of Maastricht, Maastricht, The Netherlands

**Keywords:** Mediterranean diet, Bladder cancer, Bladder cancer risk, Epidemiology

## Abstract

**Purpose:**

The role of diet in bladder carcinogenesis has yet to be established. To date most studies have investigated dietary components individually, rather than as dietary patterns, which may provide stronger evidence for any influence of diet on bladder carcinogenesis. The Mediterranean diet has been associated with many health benefits, but few studies have investigated its association with bladder cancer risk.

**Methods:**

We investigated the potential association between the Mediterranean diet score (MDS) and risk of developing bladder cancer by pooling 13 prospective cohort studies included in the BLadder cancer Epidemiology and Nutritional Determinants (BLEND) study and applying a Cox regression analysis.

**Results:**

Dietary data from 646,222 study participants, including 3639 incident bladder cancer cases, were analysed. We observed an inverse association between Mediterranean diet and bladder cancer risk (HR_high_ 0.85 [95% CI 0.77, 0.93]). When stratifying the results on non-muscle-invasive or muscle-invasive disease or sex the association remained similar and the HR estimate was consistently below 1.00 both for medium and high adherence to the Mediterranean diet. A consistent association was observed when disregarding fat or alcohol intake.

**Conclusion:**

We found evidence that adherence to the Mediterranean diet was associated with reduced risk of developing bladder cancer, suggesting a positive effect of the diet as a whole and not just one component.

## Introduction

Bladder cancer is the sixth leading cancer in the USA, with an estimated 81,190 new cases and 17,240 deaths in 2018. Over 75% of all patients are still alive after 5 years [1]. Moreover, bladder cancer has high recurrence and is the most expensive malignancy to treat, accounting for > 3% of all cancer-related medical payments in the USA [2]. At present the better established risk factors associated with developing bladder cancer include smoking, age, male sex, occupation, and to a lesser extent obesity and physical inactivity [3–5]. Since most of the metabolites of ingested food come into direct contact with the bladder mucosa, diet might also play a role in the development of bladder cancer [6].

Previous studies of diet-related bladder cancer risk factors have tended to focus on single food items [7, 8]. For example, the Multiethnic Cohort (MEC) study, which included a total of 185,885 participants and 1137 incident bladder cancer cases, reported a hazard ratio (HR) of 0.40 (95% CI 0.23–0.69) comparing highest and lowest quartiles of vegetable intake [9]. Also, the Los Angeles Bladder Cancer (case–control) Study involving 3246 participants, including 1660 cases, reported a positive association between intake of red meat (salami, pastrami and beef) and bladder cancer risk (comparing highest and lowest quintile: OR 1.33, 95% CI 1.02–1.74) [10]. Emerging evidence suggests that total dietary patterns may provide stronger evidence for diet–disease associations than individual dietary items [11].

The Mediterranean diet has been reported to be effective for preventing non-communicable diseases [12–15] and reducing overall mortality and the incidence of several cancers [16, 17]. It is generally characterized by a high consumption of fruits, vegetables, legumes and cereals, moderate-to-high consumption of fish, moderate consumption of alcohol (mostly wine), low-to-moderate consumption of milk and dairy products, and low consumption of meat and meat products [18]. The diet distinguishes itself from other dietary recommendations and indices such as the Healthy Eating Index [19], the World Cancer Research Fund and American Institute for Cancer Research (WCRF/AICR) diet recommendations [20] and the Diet Inflammatory Index [21], by its higher levels of dietary fat, mainly monounsaturated fat from olive oil, and higher alcohol consumption, mainly from wine, although alcohol is a risk factor for several cancers [22–26].

To date, few studies [27, 28] have investigated the association between Mediterranean diet and bladder cancer. The European Prospective Investigation into Cancer and Nutrition (EPIC) cohort study, including 477,312 participants (of which 1425 were incident cases), found an inverse but non-significant association comparing a high with a low Mediterranean diet score (MDS) and urothelial cell carcinoma (UCC) overall (HR 0.84 [95% CI 0.69, 1.03]), and for risk of aggressive (HR 0.88 [95% CI 0.61, 1.28]) and non-aggressive disease (HR 0.78 [95% CI 0.54, 1.14]). The association was statistically significant for current smokers (HR 0.66 [95% CI 0.47, 0.93]) [27]. Researchers from the Melbourne Collaborative Cohort Study (MCCS), which included 37,442 participants at time of recruitment (379 incident cases), reported an inverse association for both sexes between the MDS and invasive UCC (HR 0.86 [95% CI 0.74, 1.00]) [28].

Our primary aim was to build on the results of the EPIC cohort study and the MCCS, and to investigate prospectively the potential association between Mediterranean diet and the risk of developing bladder cancer, by aggregating data from 13 cohort studies in a pooled analysis using a meta-analysis approach. Our secondary aims were to examine heterogeneity in any association by sex and disease sub-type (non-muscle-invasive and muscle-invasive bladder cancer).

## Materials and methods

### Study population

Data were analysed from the Bladder cancer Epidemiology and Nutritional Determinants (BLEND) study. BLEND is a large international nutritional consortium, which included 16 cohort studies conducted in several countries. Thirteen of the 16 cohort studies had sufficient information on food items to be eligible for inclusion in our study on adherence to the Mediterranean diet and the risk of developing bladder cancer. Studies originated from centres in Denmark [29], France [30], Germany [31], Greece [32], Italy [32, 33], The Netherlands [34], Norway [35], Spain [32], Sweden [36–38] United Kingdom [39, 40], the USA [41], and Australia [42, 43].

### Data collection and coding

Details on the methodology of the BLEND consortium have been described elsewhere [44]. Briefly, the primary data from all included studies were incorporated into one dataset. All data provided were checked and converted from daily, monthly, or yearly food intake to weekly intake, and intakes by portion were also converted to intake by grams. Data on bladder cancer diagnosis were mainly ascertained by self-reported questionnaires. Dietary data, collected using food frequency questionnaires in all studies, were recoded using the Eurocode 2 food coding system [45]. In addition to information on dietary intake, the BLEND data also included study characteristics (design, method of dietary assessment, recall time of dietary intake and geographical region), participant demographics (age, sex, and ethnicity), bladder cancer pathology (non-muscle-invasive and muscle-invasive disease), and smoking status (current/former/never) all measured at baseline.

### Mediterranean diet score

To measure the degree of adherence to the Mediterranean diet, we used a nine-point scale that was constructed by Trichopoulou et al. [46]. Nine food items were included, namely, consumption of (1) cereals, (2) fruits and nuts, (3) vegetables, (4) legumes, (5) fish, (6) meat, (7) dairy products, (8) fats, and (9) alcohol/ethanol. For each component, a value of 0 or 1 was assigned using its sex-specific median for each study as a cutoff value. For the presumed beneficial components (vegetables, legumes, fruits and nuts, cereals, and fish), a value of 0 was assigned to those consuming less than the median cutoff, and a value of 1 was assigned to those consuming as much as the median cutoff or more. For the presumed detrimental components (meat and dairy products), a value of 1 was assigned to those consuming less than the median cutoff, and a value of 0 was assigned to those consuming as much as the median cutoff or more. For alcohol, a value of 1 was assigned to men consuming between 70 and 350 g per week and to women consuming between 35 and 175 g per week. We assumed that one portion of alcohol of any type contained a standard amount of 10 g of ethanol. For fat intake, we calculated the ratio of fats from plant sources to total fat and assigned a value of 0 to those consuming less than the median cutoff, and a value of 1 to those consuming as much as the median cutoff or more. We used the ratio of plant-to-total fat because we hypothesized that the effect of dietary fat may depend on its source and not solely on the quantity consumed. For example, monounsaturated fat is present in both olive oil and animal products, and by just summing up the total amount of monounsaturated fat consumed it may not take into account the potentially different biological responses related to dietary source.

The MDS ranged from 0 (minimal adherence) to 9 (maximal adherence). Scores between 0 and 3 were classified as “low adherence”, scores of 4 and 5 were classified as “medium adherence”, and scores of 6 or higher were classified as “high adherence”.

### Statistical analysis

Cox proportional hazard models using age at recruitment as the starting point on the time scale were used to calculate HRs and 95% confidence intervals (95% CI) for developing bladder cancer, comparing medium and high adherence with low adherence. The MDS was also analysed as a continuous variable (0–9). The proportional hazards assumption was examined through Schoenfeld residuals [47]. When considering all included participants, the assumption of proportional hazards was violated and therefore we compared the association between MDS score and risk of bladder cancer in all subjects younger than 70 years and in those older than 70 years to assess to what degree the HR changed over time. The Cox regression models were all adjusted for total energy intake in kilocalories (by applying a restricted cubic spline), sex and smoking status (never, former or current smoker). Furthermore, survival time was estimated by subtracting age at exit by age at entry in the cohort as *T*_0_, thereby correcting for age in the analysis and also the study sample from which the cases originated was corrected for by introducing study ID as a random effect. Analyses were stratified on sex and disease sub-type (non-muscle-invasive or muscle-invasive disease). To test for residual confounding by smoking, the association between MDS score and risk of bladder cancer was also investigated while stratifying for smoking status (ever/never).

Additionally, unstratified analyses were repeated to determine the effect of both alcohol and fats as two distinctive features of Mediterranean diet, with alterations to the estimation of the MDS in an exploratory analysis. To test the effect of alcohol on the MDS, we excluded the alcohol component from the diet score. For fats, we repeated the analysis by excluding fats from the diet score altogether and by replacing the lipid ratio (fats from plant sources divided by total fats) with only olive oil intake. All statistical analyses were performed using Stata/SE 14.2 [48].

## Results

Dietary data from 646,222 study participants, including 3639 incident cases and 642,583 non-cases were analysed. Disease sub-type was known for 2425 cases, of which 945 (39%) were muscle-invasive bladder cancer (MIBC) and 1480 (61%) were non-muscle-invasive bladder cancer (NMIBC). Compared with non-cases, bladder cancer cases were more likely to be male (74%) and to be current or former smokers (79%). Of all cases, 22% originated from Scandinavian countries, 12% from Mediterranean regions, and 42% from other countries in Western Europe. The remaining 24% of the cases were living in the USA (10%) or Australia (14%); the Australian study (MCCS) oversampled people born in Greece or Italy [42, 43] (Table 1).

**Table 1:** Characteristics of the 13 eligible studies according to subject status, sex, age, TNM stage, and smoking status

Study	Denmark (EPIC)		France (EPIC)		Germany (EPIC)		Greece (EPIC)		Italy (EPIC)		The Netherlands (EPIC)		Norway (EPIC)	
	No.	%^a^	No.	%^a^	No	%^a^	No.	%^a^	No.	%^a^	No.	%^a^	No.	%^a^
Subject status														
Total	56,005	100	64,866	100	49,457	100	25,268	100	45,204	100	37,102	100	33,856	100
Cases	411	< 1	31	< 1	218	< 1	50	< 1	192	< 1	119	< 1	24	< 1
Non-cases	55,594	> 99	64,835	> 99	49,239	> 99	25,218	> 99	45,012	> 99	36,983	> 99	33,832	> 99
Sex														
Men	26,764	48	0	0	21,551	44	10,438	41	14,084	31	9801	26	0	0
Women	29,241	52	64,866	100	27,906	56	14,830	59	31,120	69	27,301	74	33,856	100
Age														
< 50	0	0	27,158	42	23,661	48	10,715	42	21,565	48	16,161	43	21,301	63
50–59	40,996	73	26,392	41	16,978	34	5542	22	17,791	39	14,720	40	12,555	37
60–69	15,009	27	11,286	17	8817	18	6455	26	5647	13	6217	17	0	0
≥ 70	0	0	30	< 1	1	< 1	2556	10	201	< 1	4	< 1	0	0
TNM stage														
Invasive	44	24	5	12	40	26	N/A	N/A	20	20	23	20	N/A	N/A
Non-invasive	138	76	22	78	114	74	N/A	N/A	104	80	93	80	N/A	N/A
Smoking status														
Never smoker	19,624	35	45,797	71	22,658	46	14,060	56	20,540	45	14,171	38	12,057	36
Former smoker	17,070	31	13,121	20	16,386	33	4232	17	12,096	27	11,572	31	10,438	31
Current smoker	19,311	34	5948	9	10,413	21	6976	27	12,568	28	11,359	31	11,361	33
MDS														
0–3	12,595	22	30,882	48	19,758	40	6895	27	13,935	31	16,255	44	12,147	36
4–5	25,549	46	28,380	44	22,919	46	12,073	48	23,186	51	16,484	44	15,600	46
6–9	17,861	32	5604	8	6780	14	6300	25	8083	18	4363	12	6109	18

Study	Spain (EPIC)		Sweden (EPIC)		United Kingdom (EPIC)		USA (VITAL)		Netherlands (NLCS)		Australia (MCCS)^b^	
	No.	%^a^	No.	%	No.	%^a^	No.	%^a^	No.	%^a^	No.	%^a^
Subject status												
Total	40,782	100	49,328	100	75,035	100	76,433	100	5,632	100	38,263	100
Cases	154	< 1	303	< 1	250	< 1	378	< 1	940	17	520	1
Non-cases	40,628	> 99	49,025	> 99	74,785	> 99	76,055	> 99	4692	83	37,743	99
Sex												
Men	15,439	38	22,546	46	22,476	30	36,792	52	3052	54	15,798	41
Women	25,343	62	26,782	54	52,559	70	40,089	48	2580	46	22,465	59
Age												
< 50	22,824	56	19,136	39	39,461	52	0	0	0	0	12,047	32
50–59	12,936	32	16,794	34	17,049	23	35,262	46	2058	37	12,560	33
60–69	5022	12	11,150	23	12,553	17	26,685	35	3534	63	13,108	34
≥ 70	0	0	2248	4	5972	8	14,934	19	40	< 1	548	1
TNM stage												
Invasive	7	14	N/A	N/A	6	86	121	35	443	52	232	45
Non-invasive	50	86	N/A	N/A	1	14	229	65	409	48	288	55
Smoking status												
Never smoker	22,599	55	24,205	49	41,948	56	36,478	47	1848	33	22,057	58
Former smoker	7207	18	13,410	27	23,924	32	33,931	44	2018	36	11,848	31
Current smoker	10,976	27	11,713	24	9163	12	6490	9	1766	31	4358	11
MDS												
0–3	20,067	49	13,466	27	24,162	32	29,434	39	2181	39	22,326	59
4–5	17,231	42	25,798	52	29,122	39	29,194	39	2409	43	10,411	27
6–9	3484	9	10,064	21	21,751	29	15,921	22	1042	18	5314	14

*EPIC* European prospective investigation into cancer and nutrition, *NLCS* Netherlands Cohort Study, *VITAL* VITamins And Lifestyle Study, *MCCS* Melbourne Collaborative Cohort Study, *TNM stage* tumour nodes metastasis stage, *MIBC* muscle-invasive bladder cancer, *NMIBC* non-muscle-invasive bladder cancer

^a^The sum does not add up to the total, because of missing values

^b^Recruitment of the MCCS is still ongoing, therefore the presented number of participants differ from the 2016- and 2017-published numbers by Dugue et al.

The overall HR estimates for bladder cancer associated with MDS, after adjustment for total energy intake, smoking status, and sex, are presented in Table 2. A total of 6,577,179 person years, including 3581 cases, were analysed. Overall, high adherence to the Mediterranean diet was associated with a decrease in bladder cancer risk compared with low adherence (HR_high_ 0.85 [95% CI 0.77, 0.93]). A decreased bladder cancer risk was also found for medium compared with low adherence to the Mediterranean diet (HR_medium_ 0.91 [95% CI 0.85, 0.99]). In addition, an inverse linear association was found between a one-unit increase in adherence to the Mediterranean diet and risk of developing bladder cancer (HR_continuous_ 0.96 [95% CI 0.94, 0.98]). Although the proportional hazards assumption was violated, the results were similar when considering only those younger than 70 years at entry in the study (HR_high_ 0.80, [95% CI 0.72, 0.89], HR_medium_ 0.90, [95% CI 0.83, 0.98]) separately from those older than 70 years at entry in the study (HR_high_ 0.86, [95% CI 0.57, 1.29], HR_medium_ 0.82, [95% CI 0.60, 1.14]), indicating that the presented HRs in Tables 2 and 3 were probably not heavily influenced by this violation. Furthermore, residual confounding by smoking seemed minimal as the results in never smokers (HR_high_ 0.84, [95% CI 0.68, 1.04], HR_medium_ 0.84, [95% CI 0.71, 0.99]) were similar to those in ever smokers (HR_high_ 0.80, [95% CI 0.71, 0.89], HR_medium_ 0.90, [95% CI 0.83, 0.98]).

**Table 2:** Pooled HR and 95% CI for the association between adherence to the Mediterranean diet and risk of developing bladder cancer for all bladder cancer, by sex, and by disease sub-type

Diet score^a^	Both sexes			Male			Female		
	Cases/person-time^b^	Pooled HR	95% CI	Cases/person-time^a^	Pooled HR	95% CI	Cases/person-time^a^	Pooled HR	95% CI
All bladder cancer^c^									
Low (0–3)	1483/2,460,613	1.00	Reference	1082/756,521	1.00	Reference	399/1,703,192	1.00	Reference
Medium (4–5)	1479/2,868,685	0.91	0.85–0.99	1113/951,445	0.89	0.82–0.97	340/1,920,564	0.84	0.73–0.98
High (6–9)	619/1,247,881	0.85	0.77–0.93	498/462,294	0.86	0.77–0.96	149/783,160	0.90	0.74–1.10
MDS continuous	3581^d^/6,577,179	0.96	0.94–0.98	2693/2,170,260	0.95	0.93–0.98	888/4,406,918	0.96	0.92–1.00
Non-muscle-invasive									
Low (0–3)	643/2,156,174	1.00	Reference	484/652,250	1.00	Reference	176/1,449,731	1.00	Reference
Medium (4–5)	620/2,256,426	0.93	0.83–1.04	446/748,953	0.82	0.72–0.94	138/1,510,539	0.86	0.68–1.09
High (6–9)	251/933,699	0.86	0.74–0.99	212/370,334	0.87	0.74–1.03	58/614,493	0.94	0.69–1.29
MDS continuous	1514/5,346,298	0.96	0.94–0.99	1142/1,771,536	0.96	0.92–0.99	372/3,574,763	0.97	0.92–1.04
Muscle-invasive									
Low (0–3)	408/1,291,420	1.00	Reference	326/475,555	1.00	Reference	87/796,549	1.00	Reference
Medium (4–5)	355/1,427,419	0.88	0.76–1.02	279/570,121	0.80	0.68–0.95	73/850,470	0.99	0.70–1.38
High (6–9)	167/625,505	0.89	0.74–1.07	132/290,429	0.85	0.69–1.05	33/316,218	1.05	0.68–1.60
MDS continuous	930/3,344,345	0.94	0.90–0.97	737/1,336,106	0.94	0.90–0.98	193/2,008,238	0.95	0.88–1.04

^a^All results are from multivariate model adjusted for total energy intake, smoking status and sex & age at study inclusion and study sample through setting of survival time

^b^Total number of cases in adherence category may change by sex, because adherence is calculated separately in each stratum

^c^Number of cases do not add up, because of missing values on stage at diagnosis

^d^Total number of cases in analysis (3.581) lower than Table 1 (3.590) because of missing values in energy intake and/or MDS score

**Table 3:** Pooled HR and 95% CI of the analyses exploring the effects of alcohol and fats on the MDS score

MDS	Overall^a^			Fat-ratio replaced by olive oil only in MDS score^a^			No alcohol in MDS score^a^			No fat in MDS score^a^		
	Cases (person-time)	Pooled HR	95% CI	Cases (person-time)	Pooled HR	95% CI	Cases (person-time)	Pooled HR	95% CI	Cases (person-time)	Pooled HR	95% CI
Low (0–3)	1483 (2,460,613)	1.00	Reference	1478 (2,177,423)	1.00	Reference	1528 (2,705,709)	1.00	Reference	1885 (3,335,869)	1.00	Reference
Medium (4–5)	1479 (2,868,685)	0.91	0.85–0.99	1494 (2,918,929)	0.91	0.84–0.98	1405 (2,838,719)	0.93	0.86–1.00	1374 (2,618,681)	0.92	0.85–0.99
High (6–9)	619 (1,247,881)	0.85	0.77–0.93	609 (1,480,826)	0.82	0.74–0.90	396 (796,459)	0.93	0.83–1.04	322 (622,627)	0.88	0.78–0.99
MDS continuous	3581 (6,577,179)	0.96	0.94–0.98	3581 (6,577,178)	0.95	0.93–0.97	3329 (6,340,889)	0.98	0.95–1.00	3581 (6,577,178)	0.95	0.93–0.97

^a^Multivariate model adjusted for total energy intake, smoking status, sex and age at study inclusion and study sample through setting of survival time

Results remained consistently below 1.00 for non-muscle-invasive (HR_high_ 0.86 [95% CI 0.74, 0.99]) and muscle-invasive (HR_high_ 0.89 [95% CI 0.74, 1.07]) patients after stratification on disease sub-type (Table 2).

Results for men (HR_high_ 0.86 [95% CI 0.77–0.96], HR_medium_ 0.89 [95% CI 0.82, 0.97]) and women (HR_high_ 0.90 [95% CI 0.74–1.10], HR_medium_ 0.84 [95% CI 0.73, 0.98]) were comparable and in line with the overall estimates. Although total person-time was higher for women, the total number of cases was much higher for men (Table 2).When stratified on both disease sub-type and sex, HRs were consistently below 1.00, except for high compared with low adherence to the Mediterranean diet and risk of muscle-invasive disease for women (HR_high_ 1.05 [95% CI 0.68, 1.60]) (Table 2).

In the exploratory analysis, we obtained similar results after excluding either fats (HR_high_ 0.88 [95% CI 0.78, 0.99], HR_medium_ 0.92 [95% CI 0.85, 0.99]) or alcohol (HR_high_ 0.93 [95% CI 0.83, 1.04], HR_medium_ 0.93 [95% CI 0.86, 1.00]) from the diet score. Also, consistent results were found in the relation between adherence to the Mediterranean diet and bladder cancer risk when we replaced the lipid ratio (fats from plant sources divided by total fats) with olive oil intake only (HR_high_ 0.82 [95% CI 0.74, 0.90], HR_medium_ 0.91 [95% CI 0.84, 0.98]).

## Discussion

### Main findings

We investigated the association between adherence to the Mediterranean diet and bladder cancer risk and observed an overall inverse association between a high adherence to the Mediterranean diet and the risk of developing bladder cancer. Analyses stratified by sex and disease sub-type showed similar results, indicating that the association is unlikely to be confounded by factors that might differ between these subgroups.

Previously published results from studies that have investigated the association between adherence to the Mediterranean diet and the risk of developing bladder cancer are in line with our findings. Although not statistically significant, Buckland et al. [27] reported inverse associations between adherence to the Mediterranean diet and risk of overall, aggressive or non-aggressive bladder cancer for men and women. In contrast to our association between Mediterranean dietary adherence and non-muscle-invasive disease, Dugué et al. [28], based on the MCCS, found a weak inverse association between adherence to the Mediterranean diet and urothelial cell carcinoma only. It is worth mentioning that these two studies [27, 28] used different dietary fat assessment measures for the Mediterranean diet. Buckland et al. also used a different grading score for determining dietary adherence.

Despite the limited evidence for a role of the Mediterranean diet in the development of bladder cancer overall, several studies have focused on some key elements of this dietary pattern and found some beneficial effects. For example, it has been shown that the consumption of vegetables and fruits is inversely associated with the risk of bladder cancer [9, 49]. This finding is not unexpected, since both vegetables and fruits contain large quantities of polyphenols, carotenoids, and vitamins C and E, which have antioxidant functions, allowing them to prevent DNA damage by neutralizing reactive oxygen species [50, 51]. Conversely, a positive association with the risk of developing bladder cancer has been reported for high consumption of animal products, such as red and processed meats and animal proteins [52–54]. During high-temperature cooking of meat, specific substances which are known to be involved in bladder cancer carcinogenesis are formed [55]. In addition, red meat is rich in iron, which is associated with increased formation of N-nitroso compounds (NOCs). These compounds have been suggested to induce tumours in the bladder [56].

While reportedly lower in saturated and animal fats, the Mediterranean diet is associated with a higher intake of dietary fat (approximately 35% of total energy intake) usually from monounsaturated dietary fat. Another important element of the Mediterranean diet that has been studied as a single food item in the relation with bladder cancer is olive oil. Both Goulas et al. [57] and Brinkman et al. [58] showed that a higher intake of olive oil reduced bladder cancer risk. Traditionally, it has been thought that the monounsaturated fat component of olive oil was at least partly responsible for the Mediterranean diet’s health benefits but, after reviewing our sensitivity analyses using different dietary fats, this does not appear to be the case.

A possible additional explanation for a protective effect of the Mediterranean diet might be the high concentration of polyphenols in olive oil. These dietary factors are well known for their anti-oxidative and anti-inflammatory properties [59, 60]. In addition, polyphenols have been shown to have a beneficial effect on cellular function [61]. Since processes such as deregulated cell proliferation and suppressed cell death often provide a basis for tumour progression, polyphenols in olive oil may help to protect the cells of the bladder membrane against further metastasis [61]. High concentrations of polyphenols can also be found in wine, which is the main source of alcohol consumption in Mediterranean regions. Although it was expected that high concentrations of polyphenols from olive oil and wines could explain the beneficial effect of adhering to the Mediterranean diet on bladder cancer risk, it was not evident from our analyses. Therefore, more detailed analysis on polyphenols and other components of the Mediterranean diet in their relation to bladder cancer risk is needed to help explain the beneficial effect of high adherence.

Although BLEND is the largest known pooled cohort study investigating associations between adherence to the Mediterranean diet and risk of developing bladder cancer, with enough statistical power to permit detailed analyses and to detect smaller effects, it has several limitations. First, limited information was available for other possible risk factors for bladder cancer, such as body mass index, physical inactivity, socioeconomic status, and occupational exposures to carcinogenic chemicals. Adjustments for these factors could have influenced our results. Nevertheless, the current literature suggests only a small proportion of bladder cancer cases can be attributed to these factors [62–64]. The study of Buckland et al. [27] found a significantly inverse association for current smokers after stratification for smoking status. We repeated this stratified analysis using our data, and although the inverse association of a high adherence to the MDS and bladder cancer was only statistically significant in ever smokers (HR_high_ 0.80, [95% CI 0.71, 0.89]), the stratified HR estimates did not seem to differ substantially between never smokers and ever smokers.

Another limitation of our study includes potential misclassification of frequency of food consumption derived from food frequency questionnaires (FFQs), which could lead to systematic and random error when estimating adherence to the Mediterranean diet within individual studies [65]. Also, we were not able to take into account any possible changes of dietary and lifestyle habits over time, which could lead to misclassification of long-term diet. As previously reported by Dugué et al. [28], using dietary scores does not overcome the limitations inherent to FFQs, but they may help to distinguish between individuals rather than using absolute amounts of specific foods. Lastly, most of the included cohort studies used self-reported questionnaires for the ascertainment of bladder cancer diagnosis. Previous research showed that gathering diagnostic cancer information by the use of self-reported questionnaires could lead to large amounts of false negative findings, that is, cases would be falsely classified as being a non-case [66]. This could have led to underestimation of the true association.

## Conclusion

We found evidence that high adherence to the Mediterranean diet was associated with a reduced risk of developing bladder cancer. We could not isolate any particular subgroup of foods (e.g. fats, alcohol) from the MDS that provided a greater benefit over others. This may be because it describes the overall effect of the combined factors of the dietary pattern to be most protective.

## Abbreviations

BLEND: BLadder cancer Epidemiology and Nutritional Determinants
95% CI: 95% confidence interval
EPIC: European Prospective Investigation into Cancer and Nutrition
FFQ: Food Frequency Questionnaire
HR: Hazard ratio
MCCS: Melbourne Collaborative Cohort Study
MIBC: Muscle-invasive bladder cancer
NMIBC: Non-muscle-invasive bladder cancer
OR: Odds ratio
TNM stage: Tumour nodes metastasis stage
